# Treatment of the Six Year Molars, and Separation of the Teeth

**Published:** 1876-06

**Authors:** L. C. Taylor


### TREATMENT OF THE SIX YEAR MOLARS, AND SEPARATION OF THE TEETH.

BY L. C. TAYLOR.

In the May REGISTER I notice an article from the pen of Dr. R. G. Thomas, subject, Artificial Separations of the Teeth as a means of Prevention of Decay.

I have read the article with much interest, for the subject is one of vital importance to the community, and ought to be to the profession.

Dr. T. commences with the preservation of the six year molars, which is correct practice until the bicuspids make their appearance. At this point I fail to see that he has given but one course to pursue, *i. e.* to continue making the V shaped spaces during the full development of the second dentition.

Here I wish to differ a little. While I value and observe the rule he laid down, in my daily practice, in cases too far advanced to forestall and secure perfect cure by other means, yet I believe that in very many cases, we may give to our patients more and better teeth, at thirty and forty years of age, to remove the six year molars if it can be done at the right time. The space thus gained will reduce the inflammation of the gums, when the teeth are crowded, even if not irregular, and prevent the throwing off of a pernicious acid, producing rust or fungi on the labial and approximal surfaces of teeth, necessitating the separations spoken of in his good paper.

The incisors may even then be so far advanced as to require separation, if so, in all cases separate freely from the palatal side, leaving a V shaped opening, even if a filling is required.

Rarely, if ever, should separation with rubber or the wedge (when the teeth are regular) be allowed. For it is not only unnecessarily painful for the patient, but the teeth soon return to their original position only to allow the same causes to operate the second time, that operated the first, to break down the tooth substance and undermine the best filling that can be made.

The question has been asked, "If a man has been sick with a fever, would you advise him to take medicine for fear he would get sick again?" No, but I would consider him very foolish to place himself under circumstances that he knew would bring a return of the fever.

If we look into the mouths of many of our patients, we notice that one of the six year molars has been lost at the right time, not from any good judgment of the dentist, but because it ached so the patient was obliged to have it removed. In the same mouth we find the opposite molar filled, probably at the same time. What are the conditions of the teeth in that jaw? Very often ten cavities on the side where the dentist so kindly filled a poor, broken down molar, with the teeth badly crowded on that side, throwing the mesial line nearly half the diameter of the incisor toward the side where the one was lost. What is the condition of the opposite?

The teeth all free, gums healthy, and not an approximal decay can be found.

Ought we not, if possible, to ascertain the cause of such a vast difference? Is the dentist justified in allowing such cases to pass in his daily practice without careful consideration? We owe to our patients the most skillful efforts we can bring to bear to give them the best mouth possible.

The true course, in very many cases, is the one laid down in Dr. T.'s paper, yet while I endorse it heartily as far as it goes, I feel that there is something lying far back, practically of more value than the V shaped space, for the preservation of the molars and bicuspids, viz: The timely and systematic extraction of the six year molars, which makes an artificial separation. I would not extract the six year molars for every child, for if nothing needs to be done, do nothing.

There is such a variety of shapes to the bicuspid teeth, that it is very hard to lay down the law that will reach all cases. Many of them present a V shaped space sufficient to pass a pick through freely at the margin of the gum. In such cases we would be censured if we grind the enamel off until we gain a free space through to the crown, especially in young patients, for we soon find them close together, showing that what would be good practice for a patient thirty or forty years of age, will prove a failure for one sixteen.

The above suggestions are thrown out that the younger portion of the profession may be more careful in their examinations before advising their patients. A careful record of each case, with models of a large portion of the patients that come to our care, from eight to fourteen years of age, with others after the changes have all taken place, will be of value in enabling us to judge correctly as to the best course of practice.

